# Longitudinal brain atrophy distribution in advanced Parkinson's disease: What makes the difference in “cognitive status” converters?

**DOI:** 10.1002/hbm.24884

**Published:** 2019-12-02

**Authors:** Martin Gorges, Martin S. Kunz, Hans‐Peter Müller, Inga Liepelt‐Scarfone, Alexander Storch, Richard Dodel, Rüdiger Hilker‐Roggendorf, LANDSCAPE Consortium, Daniela Berg, Elke Kalbe, Heiko Braak, Kelly Del Tredici, Simon Baudrexel, Hans‐Jürgen Huppertz, Jan Kassubek

**Affiliations:** ^1^ Department of Neurology University of Ulm Ulm Germany; ^2^ German Center of Neurodegenerative Diseases and Hertie Institute for Clinical Brain Research Tübingen Germany; ^3^ Department of Neurology University of Rostock Rostock Germany; ^4^ Division of Neurodegenerative Diseases, Department of Neurology Technische Universität Dresden Dresden Germany; ^5^ German Centre for Neurodegenerative Diseases (DZNE) Rostock/Greifswald Rostock Germany; ^6^ Department of Neurology Philipps University Marburg Marburg Germany; ^7^ Department of Neuro‐Geriatrics University Clinic Essen Germany; ^8^ Klinik für Neurologie und Klinische Neurophysiologie Klinikum Vest, Knappschaftskrankenhaus Recklinghausen Recklinghausen Germany; ^9^ Department of Neurology Christian Albrecht University Kiel Germany; ^10^ Medical Psychology | Neuropsychology and Gender Studies Center for Neuropsychological Diagnostics and Intervention (CeNDI), Faculty of Medicine and University Hospital Cologne Cologne Germany; ^11^ Department of Neurology J.W. Goethe University Frankfurt/Main Germany; ^12^ Swiss Epilepsy Clinic Klinik Lengg, Zürich Switzerland

**Keywords:** atlas‐based volumetry, Braak stages, cortical thickness, LANDSCAPE study, longitudinal, magnetic resonance imaging, Parkinson's disease dementia (PDD)

## Abstract

We investigated the brain atrophy distribution pattern and rate of regional atrophy change in Parkinson's disease (PD) in association with the cognitive status to identify the morphological characteristics of conversion to mild cognitive impairment (MCI) and dementia (PDD). T1‐weighted longitudinal 3T MRI data (up to four follow‐up assessments) from neuropsychologically well‐characterized advanced PD patients (*n* = 172, 8.9 years disease duration) and healthy elderly controls (*n* = 85) enrolled in the LANDSCAPE study were longitudinally analyzed using a linear mixed effect model and atlas‐based volumetry and cortical thickness measures. At baseline, PD patients presented with cerebral atrophy and cortical thinning including striatum, temporoparietal regions, and primary/premotor cortex. The atrophy was already observed in “cognitively normal” PD patients (PD‐N) and was considerably more pronounced in cognitively impaired PD patients. Linear mixed effect modeling revealed almost similar rates of atrophy change in PD and controls. The group comparison at baseline between those PD‐N whose cognitive performance remained stable (*n* = 42) and those PD‐N patients who converted to MCI/PDD (“converter” cPD‐N, *n* = 26) indicated suggested cortical thinning in the anterior cingulate cortex in cPD‐N patients which was correlated with cognitive performance. Our results suggest that cortical brain atrophy has been already expanded in advanced PD patients without overt cognitive deficits while atrophy progression in late disease did not differ from “normal” aging regardless of the cognitive status. It appears that cortical atrophy begins early and progresses already in the initial disease stages emphasizing the need for therapeutic interventions already at disease onset.

## 1 INTRODUCTION

While there are excellent therapeutic concepts for nowadays well‐manageable motor symptoms (Chaudhuri, Odin, Antonini, & Martinez‐Martin, 2011), one of the major challenges in advanced Parkinson's disease (PD) is decreasing cognitive functioning since up to 80–83% of PD patients develop dementia (Goldman et al., 2018; Hely, Reid, Adena, Halliday, & Morris, 2008). Impaired cognition negatively impacts functioning, quality of life, caregiver burden, and health‐related costs; therapeutic options are still limited and have been highlighted as a major target for future clinical trials (Aarsland et al., 2017). Neuropathological staging of PD has shown that the Lewy body pathology is likely confined to subcortical epicenters in the preclinical and in an early symptomatic stage of PD. Later, as pathology involves the neocortices including higher association areas, individuals with PD and “normal” cognition (PD‐N) may cross a barrier of barely detectable cognitive problems and develop neuropsychologically classifiable mild cognitive impairment (PD‐MCI) and eventually the full picture of dementia (PDD) (Braak et al., 2003; Braak & Del Tredici, 2017). A large body of in vivo evidence supports the staging system (Zarei et al., 2013; Zeighami et al., 2015), although it is not without controversy (Surmeier, Obeso, & Halliday, 2017; Walsh & Selkoe, 2016). By definition, longitudinal studies from postmortem material are not possible, hence, tracing the temporal dynamics of pathology patterns in vivo together with clinical features becomes increasingly important (Fereshtehnejad, Zeighami, Dagher, & Postuma, 2017). The regional pattern of cortical brain atrophy in PD remarkably resembles the spatial distribution of cognition‐related “resting‐state” fMRI networks (Zeighami et al., 2015), and the atrophy distribution appears to be predicted by hyperconnective pathways (Yau et al., 2018). Following longitudinal studies in PD patients that demonstrated a region‐specific accelerated cortical thinning (Mak et al., 2015), we were encouraged to test the hypothesis whether the rate of volumetric changes and cortical thinning over time allows for the definition of a cognitive status‐dependent pattern in advanced PD.

Using longitudinal fully automatic atlas‐based volumetry (ABV) (Huppertz et al., 2016; Huppertz, Kröll‐Seger, Klöppel, Ganz, & Kassubek, 2010) and cortical thickness analysis of 3D MRI data (Hutton, Draganski, Ashburner, & Weiskopf, 2009; Pereira et al., 2012), we aimed to investigate the possible changes of the distribution of cortical atrophy over time in cognitively well‐characterized PD patients with a mean disease duration of about 9 years from the LANDSCAPE study cohort (Balzer‐Geldsetzer et al., 2011). Next, we investigated the atrophy distribution at the time of study entry to identify possible regional volumetric alterations associated with the conversion from normal cognition to neuropsychologically detectable cognitive deficits in PD.

## 2 METHODS

### 2.1 Study cohort

Participants included PD patients (*n* = 172) and healthy controls (*n* = 85) enrolled in the multicenter, prospective, observational LANDSCAPE study at six German centers (Balzer‐Geldsetzer et al., 2011). All participants had annual follow‐up assessments with up to five MRI scans for each individual. Demographical, clinical, and neuropsychological scales at baseline according to the previously published protocol (Balzer‐Geldsetzer et al., 2011) are summarized in Table 1.

**Table 1:** Demographic and clinical features of all participants at baseline

	PD all *n* = 172 (100%)	Controls *n* = 85	*t*‐value	*p*‐value	PD‐N *n* = 80 (46.5%)	PD‐CI *n* = 92 (53.5%)	Controls *n* = 85	*χ* ^2^	*p*‐value
Age/years	67.5 ± 8.1 [45.0–79.3]	65.1 ± 6.7 [47.0–78.7]	2.54	.012	65.9 ± 8.3 [45.0–78.7]	68.9 ± 7.7 [48.0–79.3]	65.1 ± 6.7 [47.0–78.7]	15.99	.0003
Sex (m:f)	120:52	48:37	4.44^t^	.0377^t^	56:24	64:28	48:37	4.43	.1092
Education/years	13.4 ± 2.9 [7–20]	16.1 ± 4.0 [8–30]	−5.56	<.0001	13.8 ± 2.7 [8–20]	13.0 ± 2.9 [7–20]	16.1 ± 4.0 [8–30]	33.14	<.0001
Disease duration/years	8.9 ± 4.8 [0.1–32.4]	n.a.	n.a.	n.a.	9.1 ± 5.1 [1.9–32.4]	8.8 ± 4.5 [0.1–24.1]	n.a.	0.04	.8328
CERAD/total score	89.6 ± 11.0 [41.5–106.0]	n.a.	n.a.	n.a.	95.8 ± 5.8 [78.5–106.0]	84.1 ± 11.6 [41.5–104.0]	n.a.	52.01	<.0001
DEMTECT/score (max. 18)	n.a.	16.8 ± 1.7 [13–18]	n.a.	n.a.	n.a.	n.a.	16.8 ± 1.7 [13–18]	n.a.	n.a.
PANDA/score (max. 30)	22.8 ± 5.4 [5–30]	n.a.	n.a.	n.a.	25.5 ± 3.9 [13–30]	20.5 ± 5.5 [5–29]	n.a.	38.23	<.0001
MMSE/score (max. 30)	28.1 ± 2.2 [13–30]	29.3 ± 1.0 [26–30]	−5.94	<.0001	28.9 ± 1.1 [26–30]	27.4 ± 2.6 [13–30]	29.3 ± 1.0 [26–30]	54.19	<.0001
Hoehn&Yahr/stage	2.5 ± 0.8 [1.0–5.0]	n.a.	n.a.	n.a.	2.3 ± 0.7 [1.0–4.0]	2.7 ± 0.8 [1.0–5.0]	n.a.	12.82	.0003
UPDRS III/score	21.9 ± 12.1 [3–72]	n.a.	n.a.	n.a.	18.0 ± 8.0 [5–45]	25.5 ± 13.9 [3–72]	n.a.	12.69	.0004
LEDD/(mg/d)	653.7 ± 423.9 [0.0–2015.5]	n.a.	n.a.	n.a.	654.9 ± 428.0 [0.0–2015.5]	652.6 ± 422.8 [0.0–1,584.0]	n.a.	0.01	.9243

*Note*: Values are given as mean ± *SD* [min–max]. Demographic, clinical, and neuropsychological characteristics of the total PD sample at baseline, including cognitively normal (PD‐N), mildly cognitively impaired (PD‐MCI), demented patients (PDD), and healthy controls. Data are given as mean ± std (min–max) except for gender. CERAD total score is the sum of six subscores and is sociodemographically corrected for normative data including age, education, and sex (Chandler et al., 2005). CERAD, Consortium to Establish a Registry for Alzheimer's Disease; F, female; LEDD, levodopa‐equivalent daily dose; M, male; MMSE, Mini Mental State Examination; n.a: Not available/not applicable. PANDA, Parkinson Neuropsychometric Dementia Assessment (Balzer‐Geldsetzer et al., 2011); UPDRS, Unified Parkinson's Disease Rating Scale. Unpaired two‐sample *t* test for unequal variances for continuous variables. Kruskal–Wallis ANOVA on ranks for PD‐N, PD‐CI, and healthy controls for continuous variables. Fisher's exact test was used for categorical variables.

All PD patients fulfilled strict diagnostic criteria, and all controls did not present any symptoms of neurological symptoms or other medical conditions. The LANDSCAPE study was approved by the Ethics Committee of Philipps University Marburg (approval no. 25/11). Each participating LANDSCAPE site received ethical approval from their local ethics committee and obtained detailed written and informed consent from all participants. Only subjects with 3T MRI scans (overall 597 data sets, Table S1) together with clinical and neuropsychological assessment were included in the present study.

### 2.2 Clinical and neuropsychological assessment

Demographical features and medication (summarized as levodopa‐equivalent daily dose [LEDD]) were recorded. A neuropsychological test battery was assessed by trained psychologists in six participating German institutions (Dresden, Frankfurt, Marburg, Tübingen, Ulm, and Kiel) including the German versions of the (a) Mini Mental State Examination (MMSE), (b) the Parkinson Neuropsychometric Dementia Assessment (PANDA), and (c) the Consortium to Establish a Registry for Alzheimer's Disease (CERAD) test battery. The CERAD covers the cognitive domains of (a) memory, (b) executive functions, (c) attention, (d) visuospatial functions, and (e) language. The CERAD total score was computed from four cognitive domains including 39% language, 30% learning, 11% construction, and 20% memory as previously described (Chandler et al., 2005). In particular, the subscores from Verbal Fluency (domain: language), Boston Naming Test (language), Word List Learning (learning), Constructional Praxis (construction), Word List Recall (memory), and Word List Recognition Discriminability (memory) were added up. Importantly, the resulting interim total score was further corrected for age, gender and education by adding a tabulated correction factor (see online Table E‐1 in Chandler et al., 2005).

### 2.3 Definition of cognition‐dependent subgroups

The cognitive status of PD patients was classified according to the following criteria: PD‐MCI was established according to the MCI criteria which were available at study setup (Petersen, 2004), and classification as PDD was performed according to the Movement Disorder Society Task Force guidelines (Emre et al., 2007). A patient was regarded as cognitively impaired when (a) the patient presented with cognitive impairment (either signs or symptoms reported by the patients themselves), and when (b) there was measurable poor cognitive performance, that is, ≤1.5 SD below normative mean values in at least one of the diagnostically relevant neuropsychological tests. With regard to this cut‐off score, exceptions could be made according to expert's ratings if clinicians found that clear cognitive impairment was evident despite performance above this cut‐off score (e.g., in highly educated individuals) or if a performance of a specific patient who scored below this cut‐off was still evaluated as “within normal range” by the person performing the test. Patients with cognitive impairment who (a) performed in at least one diagnostically relevant neuropsychological test in at least two cognitive domains below the normative cut‐off score, and (b) presented with significant impairment in activities of daily living according to medical history (social, occupational, or personal care) were classified as PDD (Emre et al., 2007). Cognitive status for each individual with PD was reevaluated at each follow‐up assessment (Table 2).

**Table 2:** Longitudinal demographic and clinical features of all participants

	PD all	Controls	*t*‐value	*p*‐value	PD‐N	PD‐CI	Controls	*χ* ^2^	*p*‐value
Number									
*t*_0_	172	85	n.a.	n.a.	80	92	85	n.a.	n.a.
*t*_1_ (drop‐out)	129 (−25%)	66 (−22%)	n.a.	n.a.	63 (−21%)	66 (−28%)	66 (−22%)	n.a.	n.a.
*t*_2_ (drop‐out)	54 (−68%)	37 (−57%)	n.a.	n.a.	26 (−68%)	28 (−70%)	37 (−57%)	n.a.	n.a.
*t*_3_ (drop‐out)	34 (−80%)	11 (−87%)	n.a.	n.a.	16 (−80%)	18 (−80%)	11 (−87%)	n.a.	n.a.
*t*_4_ (drop‐out)	9 (−95%)	0 (−100%)	n.a.	n.a.	6 (−93%)	3 (−97%)	0 (−100%)	n.a.	n.a.
Age/years									
*t*_0_	67.5 ± 8.1 [45.0–79.3]	65.1 ± 6.7 [47.0–78.7]	2.54	.012	65.9 ± 8.3 [45.0–78.7]	68.9 ± 7.7 [48.0–79.3]	65.1 ± 6.7 [47.0–78.7]	15.99	.0003
*t*_1_	67.8 ± 8.4 [46.4–80.1]	66.3 ± 6.6 [48.2–77.7]	1.34	.182	66.7 ± 9.0 [46.4–79.6]	68.9 ± 7.6 [49.1–80.1]	66.3 ± 6.6 [48.2–77.7]	5.52	.062
*t*_2_	67.8 ± 8.4 [47.1–81.9]	68.0 ± 6.1 [50.0–78.9]	−0.14	.888	66.2 ± 9.9 [47.1–80.5]	69.3 ± 9.4 [50.2–81.9]	68.0 ± 6.1 [50.0–78.9]	2.22	.3291
*t*_3_	68.1 ± 9.0 [48.2–81.4]	67.1 ± 5.0 [60.3–79.7]	0.44	.661	66.6 ± 9.7 [48.2–78.6]	69.4 ± 8.5 [56.8–81.4]	67.1 ± 5.0 [60.3–79.7]	1.12	.5717
*t*_4_	63.7 ± 10.8 [49.0–77.8]	–	n.a.	n.a.	62.3 ± 11.8 [49.0–77.1]	66.6 ± 10.0 [58.3–77.8]	–		
Sex (m:f)									
*t*_0_	120:52	48:37	4.443^t^	.0377^t^	56:24	64:28	48:37	4.43	.1092
*t*_1_	88:41	34:32	5.2002^t^	.0286^t^	46:17	42:24	34:32	6.38	.0412
*t*_2_	40:14	21:16	2.9795^t^	.1126^t^	18:8	22:6	21:16	3.47	.1761
*t*_3_	25:9	6:5	1.3976^t^	.2771^t^	12:4	13:5	6:5	1.4	.4975
*t*_4_	6:3	‐	n.a.	n.a.	4:2	2:1	‐	0	1
Education/years									
*t*_0_	13.4 ± 2.9 [7–20]	16.1 ± 4.0 [8–30]	−5.56	<.0001	13.8 ± 2.7 [8–20]	13.0 ± 2.9 [7–20]	16.1 ± 4.0 [8–30]	33.14	<.0001
*t*_1_	13.5 ± 2.8 [7–20]	16.3 ± 4.4 [8–30]	−4.70	<.0001	13.7 ± 2.8 [8–20]	13.3 ± 2.8 [7–20]	16.3 ± 4.4 [8–30]	21.89	<.0001
*t*_2_	13.2 ± 2.7 [8–20]	16.6 ± 4.1 [8–26]	−4.44	<.0001	13.4 ± 2.9 [8–20]	13.0 ± 2.5 [8–17]	16.6 ± 4.1 [8–26]	19.02	<.0001
*t*_3_	13.2 ± 2.7 [8–20]	18.5 ± 4.3 [13–26]	−3.86	<.0020	14.0 ± 3.1 [8–20]	12.5 ± 2.2 [9–17]	18.5 ± 4.3 [13–26]	16.11	.0003
*t*_4_	13.2 ± 2.4 [10–18]	–	n.a.	n.a.	14.0 ± 3.1 [8–20]	11.0 ± 1.4 [10–12]	–	0.6	.4386
Disease duration/years									
*t*_0_	8.9 ± 4.8 [0.1–32.4]	n.a.	n.a.	n.a.	9.1 ± 5.1 [1.9–32.4]	8.8 ± 4.5 [0.1–24.1]	n.a.	0.04	.8328
*t*_1_	9.9 ± 4.9 [1.1–33.5]	n.a.	n.a.	n.a.	10.4 ± 5.5 [2.8–33.5]	9.5 ± 4.2 [1.1–25.0]	n.a.	0.72	.3954
*t*_2_	10.8 ± 5.5 [2.4–34.5]	n.a.	n.a.	n.a.	10.8 ± 6.9 [2.4–34.5]	10.7 ± 3.7 [4.9–18.2]	n.a.	0.57	.451
*t*_3_	11.6 ± 6.3 [3.3–35.4]	n.a.	n.a.	n.a.	10.6 ± 7.6 [3.3–35.4]	12.6 ± 5.1 [5.1–22.9]	n.a.	2.7	.1004
*t*_4_	14.8 ± 10.4 [5.9–36.4]	n.a.	n.a.	n.a.	15.7 ± 11.9 [6.1–36.4]	13.2 ± 9.4 [5.9–23.8]	n.a.	0.56	.4561
CERAD/total score									
*t*_0_	89.6 ± 11.0 [41.5–106.0]	–	n.a.	n.a.	95.8 ± 5.8 [78.5–106.0]	84.1 ± 11.6 [41.5–104.0]	–	52.01	<.0001
*t*_1_	92.8 ± 9.5 [41.5–108.0]	–	n.a.	n.a.	97.2 ± 6.3 [81.0–108.0]	88.6 ± 10.2 [41.5–106.0]	–	28.08	<.0001
*t*_2_	92.6 ± 10.1 [68.5–109.0]	–	n.a.	n.a.	97.2 ± 7.1 [77.0–105.0]	88.5 ± 10.9 [68.5–109.0]	–	9.24	.0024
*t*_3_	92.5 ± 9.4 [74.0–107.0]	–	n.a.	n.a.	96.4 ± 7.6 [78.5–107.0]	89.4 ± 9.8 [74.0–105.0]	–	4.37	.0365
*t*_4_	98.6 ± 7.2 [84.0–106.0]	–	n.a.	n.a.	102.2 ± 4.4 [95.0–106.0]	97.0 ± 1.4 [96.0–98.0]	–	1.35	.2453
PANDA/score									
*t*_0_	23.1 ± 5.5 [5–30]	22.4 ± 5.9 [8–29]	0.55	.584	25.5 ± 3.9 [13–30]	20.5 ± 5.5 [5–29]	22.4 ± 5.9 [8–29]	38.23	<.0001
*t*_1_	23.2 ± 5.6 [10–30]	22.1 ± 5.9 [9–29]	0.92	.359	25.8 ± 3.8 [10–30]	20.4 ± 5.9 [8–30]	22.1 ± 5.9 [9–29]	27.94	<.0001
*t*_2_	22.6 ± 6.2 [10–30]	24.1 ± 3.4 [19–30]	−1.01	.318	26.3 ± 4.3 [12–30]	20.4 ± 5.7 [10–29]	24.1 ± 3.4 [19–30]	13.66	.0002
*t*_3_	22.5 ± 5.0 [11–30]	20.1 ± 6.9 [10–29]	0.84	.427	26.4 ± 2.5 [21–30]	24.2 ± 4.6 [15–29]	20.1 ± 6.9 [10–29]	1.34	.2468
*t*_4_	25.8 ± 4.0 [20–29]	–	n.a.	n.a.	26.8 ± 2.9 [23–30]	18.5 ± 0.7 [18–19]	–	3.53	.0603
MMSE/score									
*t*_0_	28.1 ± 2.2 [13–30]	29.3 ± 1.0 [26–30]	−5.94	<.0001	28.9 ± 1.1 [26–30]	27.4 ± 2.6 [13–30]	29.3 ± 1.0 [26–30]	54.19	<.0001
*t*_1_	28.5 ± 1.8 [21–30]	29.3 ± 0.9 [26–30]	−4.60	<.0001	29.0 ± 1.2 [25–30]	28.0 ± 2.1 [21–30]	29.3 ± 0.9 [26–30]	23.54	<.0001
*t*_2_	28.6 ± 2.0 [20–30]	29.3 ± 0.9 [26–30]	−3.08	.0030	28.6 ± 1.7 [24–30]	28.5 ± 2.3 [20–30]	29.3 ± 0.9 [26–30]	7.94	.0189
*t*_3_	28.6 ± 1.8 [24–30]	29.9 ± 0.3 [29–30]	−3.89	.0005	28.8 ± 1.8 [24–30]	28.4 ± 1.8 [25–30]	29.9 ± 0.3 [29–30]	6.36	.0415
*t*_4_	28.4 ± 2.2 [24–30]	–	n.a.	n.a.	29.0 ± 1.4 [27–30]	27.7 ± 3.2 [24–30]	‐	0.67	.557
Hoehn&Yahr/stage									
*t*_0_	2.5 ± 0.8 [1.0–5.0]	n.a.	n.a.	n.a.	2.2 ± 0.7 [1.0–4.0]	2.7 ± 0.8 [1.0–5.0]	n.a.	12.82	.0003
*t*_1_	2.3 ± 0.7 [1.0–5.0]	n.a.	n.a.	n.a.	2.3 ± 0.7 [1.0–4.0]	2.3 ± 0.7 [1.0–5.0]	n.a.	0.5	.4799
*t*_2_	2.4 ± 0.8 [1.0–4.0]	n.a.	n.a.	n.a.	2.3 ± 0.8 [1.0–4.0]	2.6 ± 0.8 [1.0–4.0]	n.a.	1.83	.1765
*t*_3_	2.5 ± 0.7 [1.0–4.0]	n.a.	n.a.	n.a.	2.3 ± 0.6 [1.0–3.0]	2.6 ± 0.7 [1.0–4.0]	n.a.	1.85	.1736
*t*_4_	2.3 ± 1.0 [1.0–4.0]	n.a.	n.a.	n.a.	2.0 ± 0.9 [1.0–3.0]	3.0 ± 1.0 [2.0–4.0]	n.a.	1.8	.1795
UPDRS III/score									
*t*_0_	21.9 ± 12.1 [3–72]	n.a.	n.a.	n.a.	18.0 ± 8.0 [5–45]	25.5 ± 13.9 [3–72]	n.a.	12.69	.0004
*t*_1_	21.3 ± 11.2 [2–60]	n.a.	n.a.	n.a.	19.4 ± 10.0 [2–58]	23.2 ± 12.1 [4–60]	n.a.	2.62	.1058
*t*_2_	22.2 ± 10.9 [3–52]	n.a.	n.a.	n.a.	20.8 ± 9.2 [3–39]	23.5 ± 12.2 [6–52]	n.a.	0.38	.539
*t*_3_	23.6 ± 10.1 [9–49]	n.a.	n.a.	n.a.	22.7 ± 8.7 [10–39]	24.5 ± 11.5 [9–49]	n.a.	0.007	.7955
*t*_4_	21.6 ± 11.0 [7–35]	n.a.	n.a.	n.a.	19.3 ± 12.5 [7–35]	26.0 ± 7.2 [18–32]	n.a.	0.61	.4367

*Note: t*_0_: baseline, *t*_*n*_ *n*th‐year follow‐up. Values are given as mean ± *SD* [min–max]. Differences between PD patients (overall cohort) and controls are computed from unpaired *t* tests for continuous variables and Fisher's exact test for categorical variables for each time point. Two right most columns refer to Kruskal–Wallis analysis of variances (ANOVA) across cognitively unimpaired PD patients (PD‐N), cognitively impaired PD patient (PD‐CI), and controls for each time point. Note that the mean age for *t*_4_ is lower than for *t*_3_ due to the study drop‐out of older patients.

### 2.4 MRI data acquisition and processing

Whole‐brain based morphological data were acquired at six study sites using a high‐resolution 3D T1‐weighted magnetization‐prepared gradient echo image (MPRAGE) sequence. Acquisition parameters largely overlapped between centers with some minor center‐specific differences as summarized in Table S1.

#### 2.4.1 Atlas‐based volumetry

Atlas‐based volumetry (ABV) was used to measure region‐specific cortical and subcortical brain volumes (Huppertz et al., 2010; Huppertz et al., 2016). All MPRAGE sequences were processed on MATLAB (R2018b, The Mathworks) using the Statistical Parametric Mapping 12 (SPM12) software (Wellcome Trust Centre for Neuroimaging, London, UK, http://www.fil.ion.ucl.ac.uk/spm) and subjected to the standardized processing pipeline for ABV (Huppertz et al., 2010). Briefly, processing includes (a) segmentation into gray matter (GM), white matter (WM), and cerebrospinal fluid (CSF) component images, (b) stereotaxic normalization into Montreal Neurological Institute (MNI) space, and (c) atlas‐based volumetry using voxel‐by‐voxel multiplication and subsequent integration of normalized modulated component images (GM, WM or CSF) with predefined masks derived from probabilistic brain atlases, as described previously (Huppertz et al., 2016). For this study, the Harvard‐Oxford atlas of subcortical structures distributed with the Oxford Centre for Functional MRI of the Brain Software Library (FSL) package (Desikan et al., 2006; Frazier et al., 2005; Goldstein et al., 2007; Makris et al., 2006) was used for subcortical structures such as hippocampus, amygdala, caudate, putamen, and thalamus, and the LONI Probabilistic Brain Atlas LPBA40 (Shattuck et al., 2008) for all other structures and compartments listed in Table 3.

**Table 3:** Volumetric results from atlas‐based volumetry (ABV) at study entry across groups

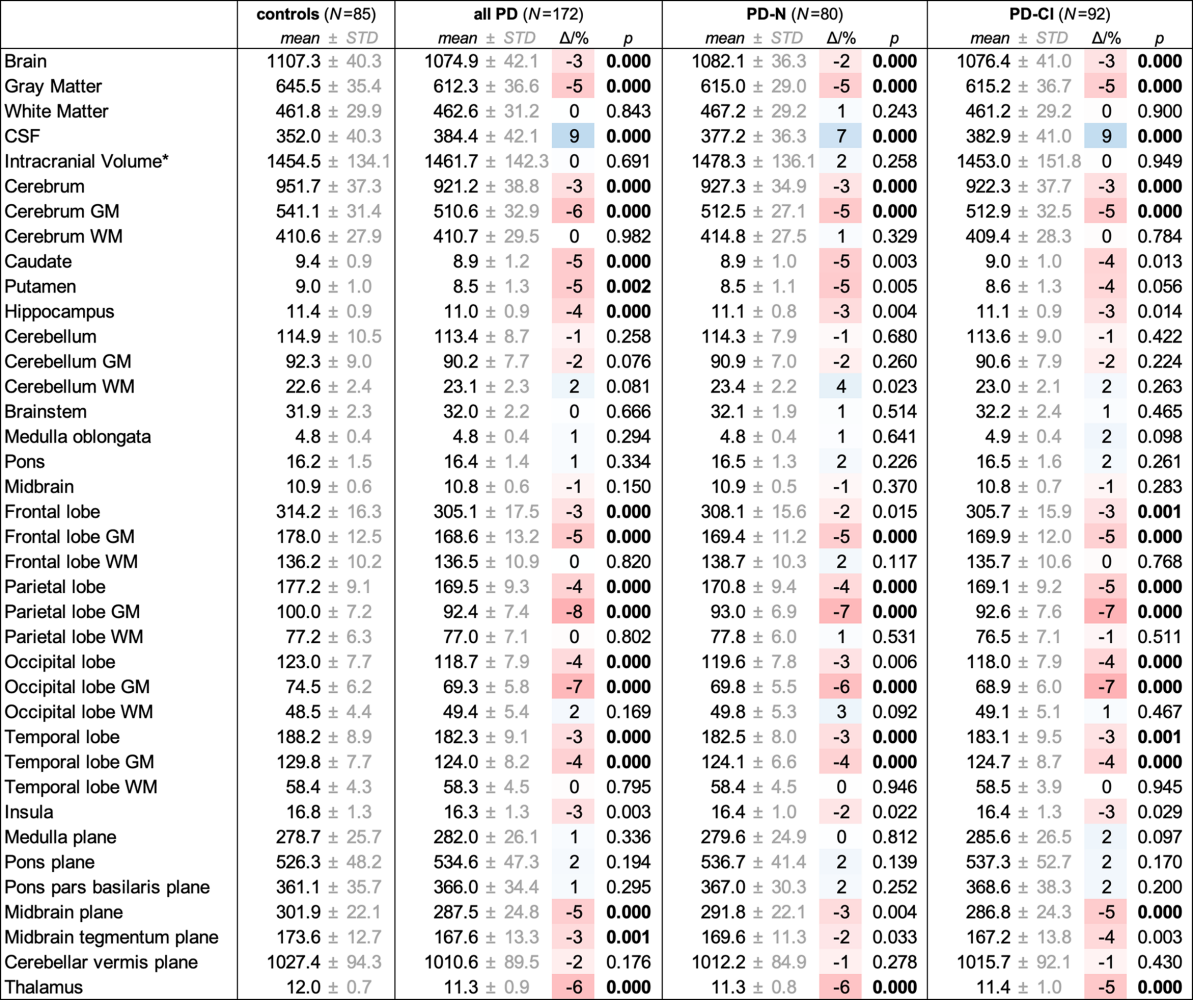

*Note*: Values are shown as volumes/cm^3^ and area/mm^2^ for planes. All values (*with the exception of the intracranial volume) are normalized to the study‐mean intracranial volume (1,459.3cm^3^) and adjusted to both the mean age (66.7 years) and mean years of education (14.3 years) of the whole study population at study entry. Deviation of means relative to controls are given as Δ/% = (*V*/*V*_controls_ − 1) × 100 and overlaid on a 3‐color scale from shades of red (volume loss) over white (no change) to shades of blue (e vacuo volume gain). The given *p*‐values resulted from group comparisons against controls; bold *p*‐values indicated statistical significance after Bonferroni correction for multiple testing. CSF, cerebrospinal fluid; GM, gray matter; WM, white matter.

#### 2.4.2 Cortical thickness

Cortical thickness was measured using the *FreeSurfer* image analysis suite (V6.0.0, http://surfer.nmr.mgh.harvard.edu/) by computing the averaged distance between the gray/white matter boundary and pial surface at each vertex on the cortical surface. Using *FreeSurfer*'s well‐established longitudinal processing pipeline (Bernal‐Rusiel et al., 2013), an unbiased subject‐specific template (Reuter & Fischl, 2011) was computed using robust, inverse consistent registration from all available MRI scans for each subject (Reuter, Rosas, & Fischl, 2010). Several processing steps including skull stripping, Talairach transformation, atlas registration as well as spherical surface maps and parcellations were performed based on the subject‐specific templates. The cerebral cortex was parcellated into 68 distinct anatomical regions for which the averaged thickness war determined. Each individual brain map was visually inspected for proper registration prior to further analysis steps.

### 2.5 Further data analysis

#### 2.5.1 Atlas‐based volumetry

Region‐wise comparisons of ABV data were performed using the MATLAB® software package (The Mathworks Inc., Natick, MA). All individual volume results *V*_*i*_ for each investigated brain area *k* were normalized to the mean intracranial volume $\bar{V^{\left(\mathrm{IC}\right)}},$ of the whole study population for baseline data using

(1) $$V_{i,k}^{\left(\mathrm{norm}\right)}=\frac{V_{i,k}}{V_{i}^{\left(\mathrm{IC}\right)}}∙\bar{V^{\left(\mathrm{IC}\right)}},$$

where $V_{i}^{\left(\mathrm{IC}\right)}$ is the intracranial (IC) volume for each individual *i*. All individually ICV‐normalized volumes $V_{i,k}^{\left(\mathrm{norm}\right)}$ were further corrected for both baseline age *x* ^(age)^ and years of education *x* ^(education)^ and adjusted to the baseline mean age ($\bar{x^{\left(\mathrm{age}\right)}}$ =66.7 years) and mean years of education ($\bar{x^{\left(\text{education}\right)}}\;$=14.3 years) for the overall study population:

(2) $$V_{i,k}^{\left(c\right)}=V_{i,k}^{\left(\mathrm{norm}\right)}-{\hat{\gamma }}_{1,k}\left(x_{i}^{\left(\mathrm{age}\right)}-\bar{x^{\left(\mathrm{age}\right)}}\;\right)-{\hat{\gamma }}_{2,k}\left(x_{i}^{\left(\text{education}\right)}-\bar{x^{\left(\text{education}\right)}}\right)$$

where $V_{i,k}^{\left(c\right)}$ denotes the ICV‐normalized and age‐ and education‐corrected volume results. The slope estimates ${\hat{\gamma }}_{i}$ resulted from solving a general linear model for all baseline data of the healthy controls:

(3) $$V_{k}^{\left(\mathrm{norm}\right)}=X\gamma_{k}+{ɛ}_{k}=\left[1\;x^{\left(\mathrm{age}\right)}\;x^{\left(\text{education}\right)}\right]\left(\begin{array}{c} \gamma_{0,k}\\ \gamma_{1,k}\\ \gamma_{2,k} \end{array}\right)+{ɛ}_{k},$$

where ***1*** is an all‐ones *n* × 1 vector, $V_{k}^{\left(\mathrm{norm}\right)}\in {\mathbb{R}}^{n}\;$, ***X*** ∈ *ℝ*^*n* × 3^ , ***ɛ***_*k*_ ∈ *ℝ*^*n*^ is the error term, *n* is the number of individuals, and *k* denotes the brain area.

#### 2.5.2 Vertex‐wise cortical thickness analysis

Whole‐brain based cortical thickness outcomes were studied using a vertex‐wise analysis pipeline implemented in *FreeSurfer* using age and years of education as covariates in general linear model. The reconstructed data sets for each subject were deformed on an average anatomical surface and blurred with a 10 mm full‐width‐at‐half‐maximum Gaussian smoothing filter (Walhout et al., 2015). Correction for multiple comparisons was performed using a Monte Carlo simulation (10,000 iterations) for a cluster‐wise correction threshold of *p* = .05 and a vertex‐wise threshold of *p* = .001.

#### 2.5.3 Region‐of‐interest‐based thickness analysis

Region‐of‐interest analysis of cortical thickness measures was performed by normalizing to the mean cortical thickness (Walhout et al., 2015) by replacing *V* ^(IC)^ with the mean cortical thickness in Equation (1). Correction for both age and years of education was applied using a general linear model according to Equations (2) and (3). The cortical thickness values for each region including “mean cortical thickness” were arithmetically averaged for both hemispheres resulting in an average value for each region and “mean cortical thickness.” Mean cortical thickness was studied by using age, years of education, and sex as a covariate according to a general linear model as provided in Equations (2) and (3). Corrected thickness measures were arithmetically averaged for both hemispheres.

### 2.6 Cross‐sectional statistical testing

Statistical data analysis of sociodemographic data, ABV‐based volumes, and region‐of‐interest‐based cortical thickness values was performed using the MATLAB®‐based Statistics Toolbox (The MathWorks, Inc., Natick, MA). Group difference for time points (e.g., baseline, follow‐up one, etc.) were analyzed using Fisher's exact text for categorical variables and unpaired *t* tests for continuous variables. Kruskal–Wallis analysis of variances on ranks was applied to test differences between three or more groups, followed by post hoc unpaired *t* tests in the event of significance. Lillifors test was applied to test for normal distribution. All correlations were computed using Spearman rank order correlation coefficient. Bonferroni correction for multiple testing was conducted when statistical contrasts were not driven by a specific hypothesis. All tests were two‐sided and *p* < .05 were considered significant.

### 2.7 Longitudinal data analysis

The group‐time trajectories of volumes and thickness measures from longitudinal MRI data were investigated in order to assess the group‐specific atrophy rate over time. In our observational study like in most longitudinal studies with large numbers of increasingly handicapped patients, several individuals dropped out during the course of evaluation (Figure S1 and Table 2) due to withdrawal of consent (8.0%), death (6.7%), early termination (5.5%), incompliance with the protocol (3.2%), or were lost to follow‐up (20.2%), possibly caused by progressive physical impairment and pronounced cognitive challenges. The reason for drop out was not documented for 56.4% of the PD patients. The unbalanced number of individuals at each point of investigation requires appropriate data modeling: the linear mixed effect (LME) models are a versatile class of complex models that properly capture individuals with different numbers of measurements over time (Fitzmaurice & Ravichandran, 2008). Both, volumetric and cortical thickness changes over time were analyzed using the LME approach.

#### 2.7.1 Exploring longitudinal data

Longitudinal data analysis requires an exploratory investigation of the temporal trajectories with possible contribution of variables and linear and nonlinear trends. The locally weighted scatterplot smoothing (LOWESS) methods (Cleveland, 1979) are a powerful approach for graphical exploratory analysis that generates a smoothed trajectory by centering a sliding window of fixed width at each sampling point and iteratively fitting a straight line to the data points within the window by means of weighted least squares. The LOWESS estimate is a readout from the fitted regression line for each time point. In the present study, the amount of smoothing based on the properties of the measurements, that is, the fraction of the total number of data points within the sliding window, was set to *f* = 0.8. The order of polynomial that is locally fit to each point of the scatterplot was set to *d* = 1, a tricubic weight function *W* for weighted regression fit was used, and the number of iterations for the robust fitting procedure was set to *t* = 2.

#### 2.7.2 Linear mixed effect modeling

LME models allow modeling of unbalanced responses of interest (i.e., unequal number of sampling points over time for each individual) over time. LME models compute the overall mean response ***y***_*i*_ as a linear combination of the population‐based mean response (“fixed‐effects”) and individual‐specific mean response trajectories over time (“random‐effects”) (Bernal‐Rusiel et al., 2013):

(4) $$y_{i}^{\left(k\right)}=X_{i}^{\left(k\right)}\beta^{\left(k\right)}+Z_{i}^{\left(k\right)}b_{i}^{\left(k\right)}+\varepsilon_{i}^{\left(k\right)}$$

where $y_{i}^{\left(k\right)}\in {\mathbb{R}}^{m_{i}}$ is the outcome of the *m*_*i*_ longitudinal measurements for individual *i* and brain region *k* with respect to the normalized volumetric ($V_{i,k}^{\left(\mathrm{norm}\right)}$) or thickness data ($T_{i,k}^{\left(\mathrm{norm}\right)}$) obtained from Equation (1). The volume values were normalized to the mean ICV and the regional cortical thickness values were normalized to the “mean cortical thickness” prior to subjecting the respective values to the LME model. $X_{i}^{\left(k\right)}\in {\mathbb{R}}^{m_{i}\times p}$ denotes the fixed effects design matrix (including covariates such as age), $Z_{i}^{\left(k\right)}\in {\mathbb{R}}^{m_{i}\times q},q\leq p$ the random effects design matrix, and $\varepsilon_{i}^{\left(k\right)}\in {\mathbb{R}}^{m_{i}}$ is the error term. The index *i* indicated the *i*th individual and the index *k* denotes the *k*th brain region of interest. The unknown model parameters ***β***^(*k*)^ ∈ *ℝ*^*p*^ and $b_{i}^{\left(k\right)}\in {\mathbb{R}}^{q}$ are to be estimated.

Unless specified otherwise, the following independent variables were used to create the fixed‐effect design matrix in Equation (4):

(5) $$X_{i}^{\left(k\right)}=\left[1_{i}\;t_{i}\;G_{i}^{\left(1\right)}\;\begin{array}{ccc} t_{i}∙G_{i}^{\left(1\right)} & G_{i}^{\left(2\right)} & t_{i}∙G_{i}^{\left(2\right)} \end{array}\;\begin{array}{cc} x_{i}^{\left(\mathrm{age}\right)} & x_{i}^{\left(\text{education}\right)} \end{array}\right]$$

where $X_{i}^{\left(k\right)}\in {\mathbb{R}}^{m_{i}\times 8},$ **1** is an all‐ones *m*_*i*_×1 vector, $t_{i}\in {\mathbb{R}}^{m_{i}}$ is the time from baseline, $G_{i}^{\left(1\right)}\in {\left\{0;1\right\}}^{m_{i}}$ is a binary group indicator variable for Group 1, that is, PD‐N patients, which is 1 if the individual is a member of Group 1 and 0 otherwise, $t_{i}∙G_{i}^{\left(1\right)}\in {\mathbb{R}}^{m_{i}}$ is the interaction between Group 1 membership and time from baseline, $G_{i}^{\left(2\right)}\in {\left\{0;1\right\}}^{m_{i}}$ is a binary group indicator variable for Group 2, that is, PD‐CI patients, which is 1 if the individual is a member of Group 2 and 0 otherwise (note that controls define the reference group), $t_{i}∙G_{i}^{\left(2\right)}\in {\mathbb{R}}^{m_{i}}$ is the interaction between Group 2 membership and time from baseline, $x_{i}^{\left(\mathrm{age}\right)}\in {\mathbb{R}}^{m_{i}}$ is the individual's age at enrollment, and $x_{i}^{\left(\text{education}\right)}\in {\mathbb{R}}^{m_{i}}$ is the individual's years of education. The index *i* specifies the *i*th individual, *m*_*i*_ is the number of longitudinal measurements for each individual, and *k* denotes the brain area. The effect of age is assumed to be negligibly small under 60 years (Hedman, van Haren, Schnack, Kahn, & Hulshoff Pol, 2012) but significant over 60 years. This nonlinear effect of age can be captured with the following piecewise linear model

(6) $$x_{i,j}^{\left(\mathrm{age}\right)}=z_{i,j}^{\left(\mathrm{age}\right)}\cdot H\left(z_{i,j}^{\left(\mathrm{age}\right)}\cdot 60a∙\right)$$

where $z_{i,j}^{\left(\mathrm{age}\right)}$ is the age for the *i*th individual for the *j*th measurement, and *H*(*x*) denotes the Heaviside step function which is one for *x* > 0 and zero otherwise.

For modeling random effects in Equation (4), both intercept and time from baseline were included for each individual in the random‐effects design matrix by assuming different (co)variances (i.e., compound symmetry is assumed not to hold for volumes/thickness values in the present study):

(7) $$Z_{i}^{\left(k\right)}=\left[1_{i}t_{i}\;\right]$$

where $Z_{i}^{\left(k\right)}\in {\mathbb{R}}^{m_{i}\times 2},$ **1** is an all‐ones *m*_*i*_×1 vector, $t_{i}\in {\mathbb{R}}^{m_{i}}\;$.

For the longitudinal analysis of atrophy progression, we obtained $\beta_{0}^{\left(k\right)}\ldots \;\beta_{7}^{\left(k\right)}$ regression parameters from the LME model with $\beta_{1}^{\left(k\right)}$, $\beta_{3}^{\left(k\right)}$, and $\beta_{5}^{\left(k\right)}$ indicating the slopes (time‐varying volumetric and thickness changes) for controls, PD‐N, and PD‐CI patients, respectively. The estimated model parameters including the covariance matrix were subjected to hypothesis testing using *F* statistics. For comparing rate of changes between groups, the null hypothesis can be expressed as *β*_3_ = *β*_5_ = 0 resulting in the corresponding contrast matrix

(8) $$L=\left[\begin{array}{c} 0\\ 0 \end{array}\begin{array}{c} \;0\;\\ 0 \end{array}\begin{array}{c} 0\\ 0 \end{array}\begin{array}{c} \;1\\ \;0 \end{array}\begin{array}{c} 0\\ \;0\; \end{array}\begin{array}{c} 0\\ 1 \end{array}\begin{array}{c} \;0\\ \;0 \end{array}\begin{array}{c} \;0\\ \;0 \end{array}\right].$$

In other words, the null hypothesis is true if the slopes (i.e., rates of changes) are the same for each group (i.e., controls, PD‐N, PD‐CI).

## 3 RESULTS

### 3.1 Atrophy distribution in advanced PD

First, we investigated volumetric changes and cortical thickness in PD patients and according subgroups PD‐N and PD‐CI in relation to healthy controls in order to quantify the atrophy distribution at study entry.

#### 3.1.1 Participants

Participants in the present study encompassed PD patients (*n* = 172) and healthy controls (*n* = 85) at study entry. With a mean disease duration of 8.9 ± 4.8 years, the PD cohort represented an advanced disease state; 80 were neuropsychologically classified as having normal cognition (PD‐N), 77 as PD‐MCI, and 15 as PDD. The PD‐MCI group and PDD group were merged as cognitively impaired PD patients (PD‐CI, *n* = 92) due to the underpowered PDD cohort. Groups (PD‐N, PD‐CI, controls) significantly differed in age (post hoc *t* test: *p* = .24 for PD‐N vs. controls; *p* < .001 for PD‐CI vs. controls; *p* = .01 for PD‐N vs. PD‐CI), years of education (post hoc *t* test: *p* < .001 for PD‐N vs. controls; *p* < .001 for PD‐CI vs. controls; *p* = .037 for PD‐N vs. PD‐CI), but not in sex. Overall cognitive performance as measured by the sociodemographically corrected CERAD total score presented a marked gradient from PD‐N patients (score 96 ± 6) to PD‐CI (score 84 ± 12, 12% loss, *t* = 8.3, *p* < .0001). Physical disability was less in PD‐N (Hoehn & Yahr stage 2.2 ± 0.7; UPDRS III 18 ± 8) than in PD‐CI (Hoehn & Yahr stage 2.7 ± 0.8, *t* = −3.84, *p* = .0002; UPDRS III 26 ± 14, *t* = −4.33, *p* < .0001). There was no significant difference in years of disease duration and levodopa equivalent daily dose (LEDD). Baseline characteristics including statistical contrasts are summarized in Table 1.

#### 3.1.2 Volumetric changes

ABV‐based volume measures were normally distributed as per Lilliefors tests and subjected to unpaired *t* test. The global brain volumes were moderately decreased by about 3% (*p* < .0001) in the overall cohort of PD patients compared to controls, resulting from gray matter loss (−5%, *p* < .0001) whereas white matter volume was similar in PD patients and controls (*p* = .843). Frontal, parietal, occipital, and temporal lobes including the hippocampus were reduced in PD compared to controls (−3% to −4%, *p* < .0001) again due to gray matter loss (*p* < .0001) but not volume reduction in white matter (*p* = .169). The striatum including the caudate (−5%, *p* < .0003) and putamen (−5%, *p* < .0016), and the thalamus (−6%, *p* < .0001), and the midbrain plane (−5%, *p* < .0001) also presented volume reductions in PD compared to controls.

Compared to controls, global brain atrophy was already present in PD‐N patients (−2%, *p* < .0001) and was slightly more pronounced in PD‐CI patients (−3%, *p* < .0001). Volume reduction for the frontal and occipital lobes and midbrain in PD‐N (vs. controls) did not reach significance after multiple comparison correction. In PD‐CI (vs. controls), the slightly more pronounced volume reduction reached significance for the midbrain (−5%, *p* = .0001), the frontal (−3%, *p* < .0001), and occipital lobe (−4%, *p* = .0001). The volumes of the hippocampus and striatum were reduced by 3–5% in both PD‐N and PD‐CI but the volume loss did not reach significance after correction for multiple testing. Overall, marked volume loss was present already in PD‐N patients and the atrophy was, if ever, mildly more pronounced in PD‐CI patients. All ABV results from baseline measurement are summarized in Table 3.

#### 3.1.3 Changes in cortical thickness

**Figure 1:**
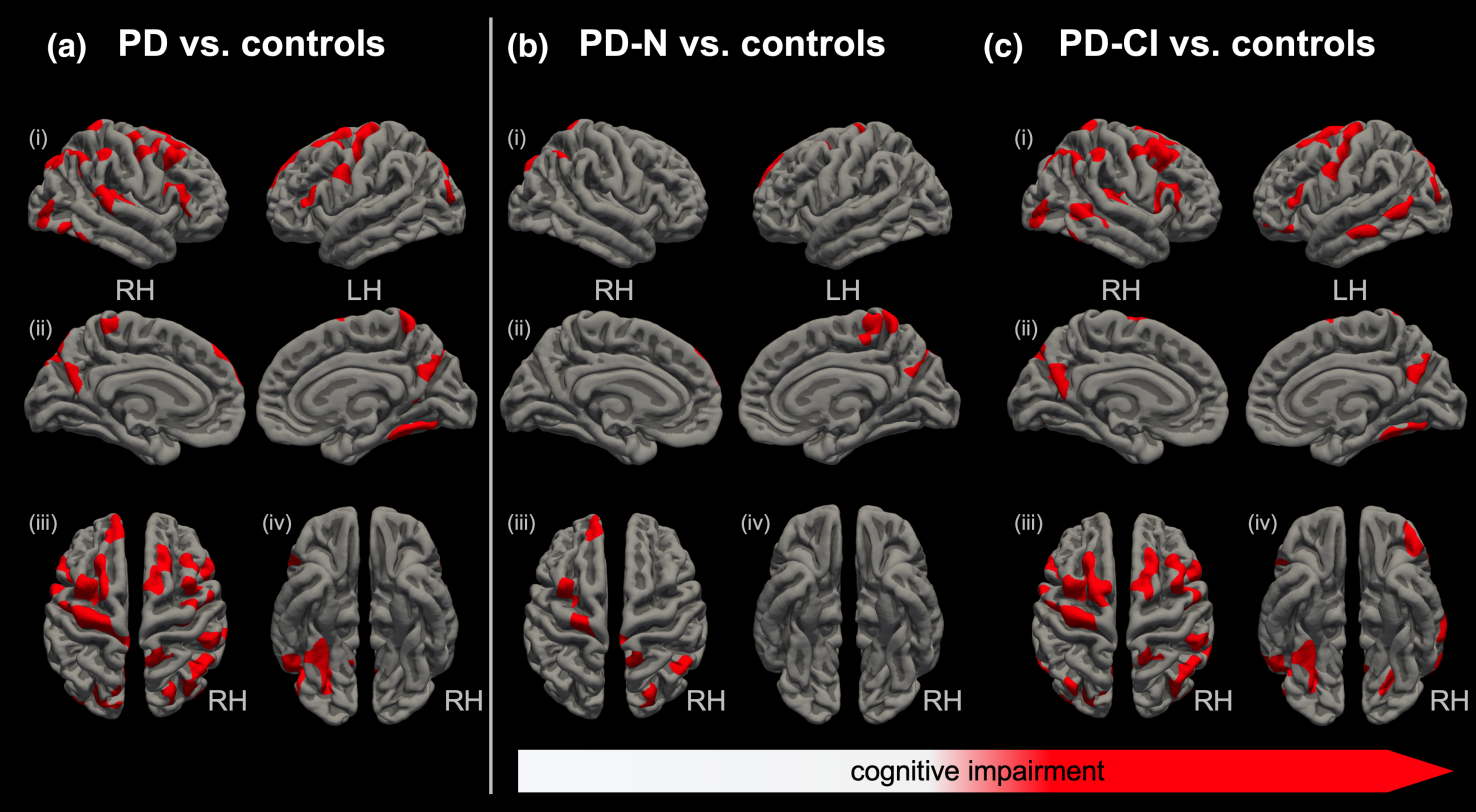
Regional differences in cortical thickness at study entry: The whole brain‐based vertex‐wise analysis for (a) the overall study cohort and (b, c) in dependence of the cognitive status. Group comparisons are illustrated for healthy controls (*n* = 76) against (a) all PD patients (*n* = 136), (b) cognitively unimpaired PD patients (PD‐N, *n* = 57), and (c) cognitively impaired PD patients (PD‐CI, *n* = 79). All shown clusters are corrected for multiple comparisons using Monte Carlo simulation at a cluster‐wise threshold of *p* < .05. Results are projected on standard pial surface views from (i) lateral (ii) medial, (iii) dorsal, and (iv) ventral perspective. RH, right hemisphere; LH, left hemisphere

Vertex‐wise whole‐brain analysis of cortical thickness confirmed the ABV‐based results of cortical involvement of PD patients at baseline. As shown in Figure 1a, frontal, occipital, temporal and parietal lobes demonstrated a widely distributed pattern of significant regional cortical thinning including large parts of the primary and premotor cortices. Cortical thinning was already observed in PD‐N patients compared to controls (Figure 1b), mainly involving the primary motor and premotor areas with mild thinning in the occipital and frontal lobe. A more pronounced pattern of cortical thinning was observed in PD‐CI patients as compared to controls (Figure 1c): here, thinning was demonstrated to a larger extent in areas that were already thinned in PD‐N patients (vs. controls). Cortical thinning was also demonstrated in the parietal and frontal lobe.

### 3.2 Comparing rate of atrophy in advanced PD patients

#### 3.2.1 Participants

From the overall study cohort (172 PD patients, 85 healthy controls), 129 PD and 85 controls had one‐year (1.1 years on average for both groups), 54 PD and 37 controls had two‐year (2.4 years on average for PD, 2.6 years for controls), 34 PD and 11 controls had three‐year (3.2 years on average for both groups), and nine PD patients had four‐year (3.9 years on average) follow‐up assessment post enrollment. The study drop‐out was similar for PD‐N, PD‐CI, and controls for each time point of assessment. Kruskal–Wallis tests revealed differences in age and sex (for baseline and 1‐year follow‐up) and years of education (for all time points), whereas disease duration did not differ. Cognitive performance scores (MMSE, PANDA, CERAD total score) revealed a marked difference between PD‐N and PD‐CI with poor cognitive performance in cognitively impaired PD patients. All longitudinal clinical and sociodemographic data including statistical contrasts are summarized in Table 2.

#### 3.2.2 Atrophy progression according to volumetric and thickness measures

**Figure 2:**
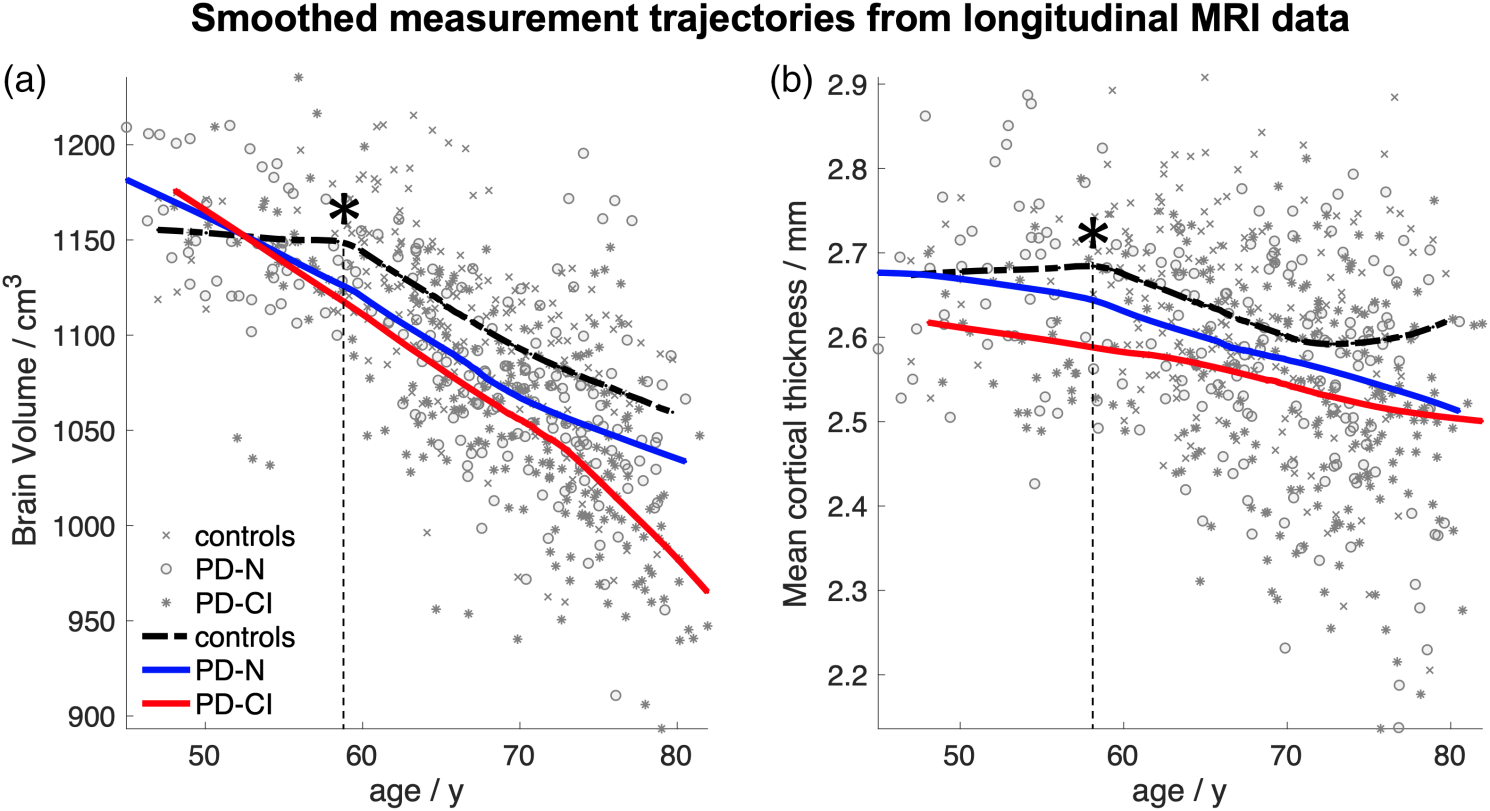
Locally weighted scatterplot smoothing (LOWESS) plot: Smoothed trajectory (amount of smoothing *f* = 0.8) for (a) the brain volume and (b) mean cortical thickness over time shown for healthy controls (dashed black line), cognitively normal PD patients (PD‐N, solid blue), and cognitively impaired PD patients (PD‐CI, solid red). The cognitive status was based on neuropsychological assessment at baseline. The LOWESS plot indicated (a) a steady atrophy progression of approximately 5cm^3^ per year (~0.5% per year) for the overall brain volume in healthy elderly over about 60 years (*) and for patients independent of age. (b) The rate of cortical thinning for mean cortical thickness in healthy elderly controls (approximately 7.3 μm per year over about 60 years of age *) and PD patients without (PD‐N) and with cognitive deficits (PD‐CI)

The main aim of the present longitudinal study was to investigate the atrophy progression rate in advanced PD patients depending on the cognitive status. Longitudinal volumetric and thickness measures from PD‐N and PD‐CI patients relative to controls were studied using LOWESS plots and subjected to an appropriate LME model. Explorative comparison of volumetric loss across groups over time using LOWESS plots suggested nonlinear trajectories as representatively shown for the overall brain volume (Figure 2a) and mean thickness (Figure 2b). Visual inspection revealed an almost constant and negligibly small volume and thickness loss for the investigated volumes and thickness measures in elderly controls before 60 years of age. Figure 2a representatively illustrates a steady atrophy progression in “normal” aging of about −5 cm^3^ per year for healthy individuals over 60 years of age. This finding of gradually accelerated volume loss of about *q*(*t =* 65a) ≈ 0.5% per year is consistent with meta analyses of brain volume changes across the lifespan in normal aging (Hedman et al., 2012). The smoothed volume trajectories may further indicate that the rate of atrophy change in PD‐CI patients (as compared with both PD‐N patients and controls) is more prominent beyond 70 years of age. However, this has to be regarded as an effect of age because PD‐CI patients are significantly older (vs. PD‐N, *p* < .001; vs. controls *p* < .001) and the LME model indicated no significant effects for the rates of atrophy change (*F* = 2.8, *DF*_1_ = 2, *DF*_2_ = 238, *p* = .06) for subjects over 70 years of age when not using age as a covariate.

**Figure 3:**
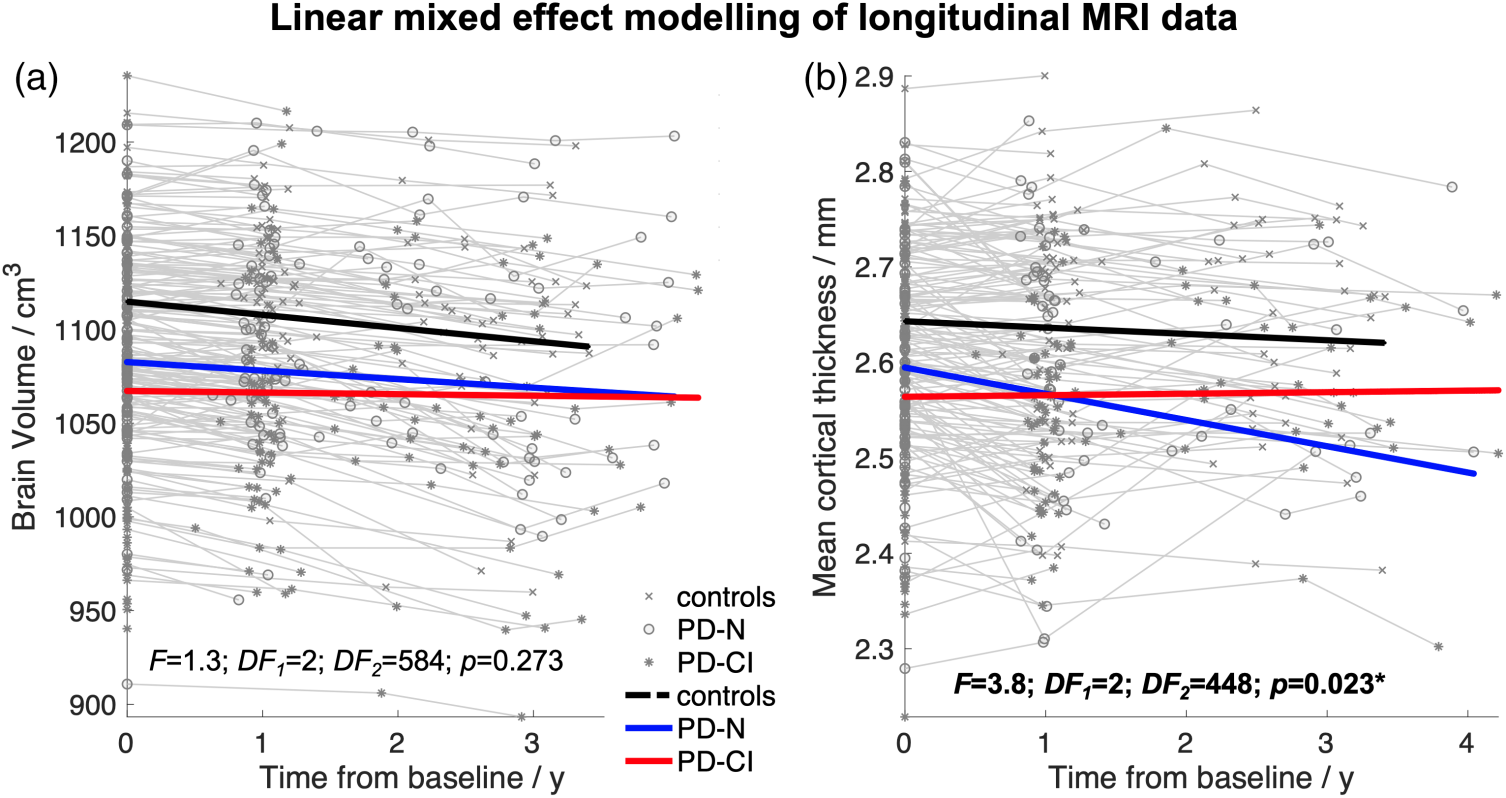
Atrophy progression in PD patients depending on the cognitive status: Linear fits of the linear mixed effect (LME) modeled data for controls (black line), cognitively unimpaired PD patients (PD‐N), and cognitively impaired PD patients (PD‐CI). The cognitive status was based on neuropsychological assessment at baseline. (a) The LME model indicated no statistical difference in atrophy progression rate for the overall brain volume across groups (*F* = 1.3; *p* = .273). (b) LME fits indicated a weak but significant effect for mean cortical thickness across groups (*F* = 3.8; *p* = .023) due to the faster cortical thinning in PD‐N patients. (a, b) The LME fits are overlaid on the individual measurement trajectories (“spaghetti plot”). DF, degrees of freedom

As shown for the brain volume in “normal” aging (Figure 2a), a steady regional cortical thinning as representatively illustrated for mean cortical thickness (Figure 2b) is obvious for individuals over about 60 years. This nonlinear effect of age was captured by using a piecewise model for age within the LME framework. For all volume data as obtained from ABV and region‐of‐interest‐based thickness values (averaged for both hemispheres), we tested the hypothesis as follows: is there any difference in rate of atrophy among PD‐N and PD‐CI patients relative to controls? In other words, the null hypothesis is true if the slopes (i.e., rates of changes) are equal for each group (i.e., controls, PD‐N, PD‐CI). Interference statistics applied to the LME model revealed no significant effects as exemplified for the brain volume change (Figure 3a) with an exception of the mean cortical thickness (*F* = 3.8, *DF*_1_ = 2, *DF*_2_ = 448, *p* = .023) indicating a mildly accelerated mean cortical thinning in PD‐N patients of −28 μm per year (as compared to PD‐CI patients, 2 μm/a, *F* = 7.1, *DF*_1_ = 2, *DF*_2_ = 448, *p* = .008) (Figure 3b).

In the interest of comparability with previous studies (Mak et al., 2015; Yau et al., 2018), longitudinal thickness analysis at 1‐year follow‐up was performed and indicated an almost similar rate of mean cortical thinning by about −30 μm per year across groups, that is, PD‐N (*n* = 68) and PD‐CI patients (*n* = 61) compared to healthy controls (*n* = 66), as indicated by Kruskal–Wallis analysis on ranks (*χ* ^2^ = 0.68, *p* = .711). The use of a general linear model (GLM) according to Freesurfer's longitudinal processing pipeline (Reuter, Schmansky, Rosas, & Fischl, 2012) resulted in no significant differences in region‐specific rate of cortical thickness changes at 1‐year follow‐up between PD‐patients (*n* = 127) and controls (*n* = 66).

Overall, the longitudinal analyses of volumetric and cortical thickness measures indicated an almost identical atrophy progression in advanced PD patients relative to “normal” aging regardless of whether patients were neuropsychologically classified as cognitively unimpaired or impaired.

### 3.3 Distribution of atrophy in “converters”

#### 3.3.1 Participants

We finally compared the atrophy distribution at study entry between cognitively normal PD patients who maintained their “normal” cognitive status throughout the observational period and those who converted to either MCI or PDD. Follow‐up assessment was available for 68 PD‐N patients at enrollment and from these individuals, 42 (62%, nPD‐N) maintained “normal” cognitive performance throughout the study, whereas 26 (38%, cPD‐N) converted to either MCI (*n* = 25; 96%) or PDD (*n* = 1; 4%). Mean time for conversion was 1.4 years (±0.1 years, range 0.7–3.0 years) after a mean disease duration of 10.9 years (±4.3 years, range 4.8–20.6 years). One of the cPD‐N patients was classified as MCI after 8 months (17.1 years of disease duration) and converted to PDD after 3.6 years (20.7 years of duration). The nPD‐N and cPD‐N groups did not significantly differ in age, sex, years of education, UPDRS III, Hoehn & Yahr, and disease duration for all assessments. The sociodemographically corrected CERAD total scores were similar in nPD‐N and cPD‐N at baseline scan but significantly differed at 1‐year follow‐up assessment (*t* = 2.7, *p* < .0092), whereas PANDA and MMSE score were similar between nPD‐N and cPD‐N groups for each time point of assessment. Note that the CERAD total score was not different for the 2‐year and 3‐year follow‐ups due to the drop‐out number of more than 58% and more than 69%, respectively. The study drop‐out was about the same for nPD‐N and cPD‐N. All characteristics of “converters” and “nonconverters” as compared to controls are summarized in Table 4. There were no significant volumetric changes for all investigated regions between nPD‐N and cPD‐N at enrollment (Table 5).

**Table 4:** Longitudinal demographic and clinical measures of “cognitive” converters

	Controls	nPD‐N	cPD‐CI	*χ* ^2^	*p*‐value*	*t*‐value**	*p*‐value**
Number							
*t*_0_	85	42	26	n.a.	n.a.	n.a.	n.a.
*t*_1_ (drop‐out)	66 (−22%)	42 (−0%)	26 (−0%)	n.a.	n.a.	n.a.	n.a.
*t*_2_ (drop‐out)	37 (−57%)	17 (−60%)	11 (−58%)	n.a.	n.a.	n.a.	n.a.
*t*_3_ (drop‐out)	11 (−87%)	10 (−76%)	8 (−69%)	n.a.	n.a.	n.a.	n.a.
*t*_4_ (drop‐out)	0 (−100%)	5 (−88%)	1 (−96%)	n.a.	n.a.	n.a.	n.a.
Age/years							
*t*_0_	65.1 ± 6.7 [47.0–78.7]	65.0 ± 9.5 [45.0–78.1]	67.8 ± 5.7 [58.4–78.7]	2.80	0.246	−1.49	0.140
*t*_1_	66.3 ± 6.6 [48.2–77.7]	66.1 ± 9.4 [46.4–79.0]	68.8 ± 5.8 [59.4–79.6]	0.73	0.693	−1.46	0.150
*t*_2_	68.0 ± 6.1 [50.0–78.9]	65.4 ± 10.2 [47.1–78.7]	71.1 ± 6.1 [63.4–80.5]	0.35	0.838	−1.88	0.071
*t*_3_	67.1 ± 5.0 [60.3–79.7]	65.6 ± 10.6 [48.2–76.9]	70.1 ± 5.4 [64.4–81.4]	20.57	0.752	−1.17	0.262
*t*_4_	‐	60.0 ± 11.6 [49.0–77.1]	73.9	0.77	0.380	−2.67	n.a.
Sex (m:f)							
*t*_0_	48:37	30:12	19:7	3.97	0.137	0.022	0.883
*t*_1_	34:32	30:12	19:7	6.01	0.494	0.022	0.883
*t*_2_	21:16	13:4	8:3	2.36	0.307	0.049	0.823
*t*_3_	6:5	7:3	6:2	0.99	0.609	0.055	0.814
*t*_4_	–	3:2	1:0	n.a.	n.a.	n.a.	n.a.
Education/years							
*t*_0_	16.1 ± 4.0 [8–30]	14.0 ± 2.9 [8–20]	14.2 ± 2.4 [11–19]	10.44	0.0054	−0.26	0.797
*t*_1_	16.3 ± 4.4 [8–30]	14.0 ± 2.9 [8–20]	14.2 ± 2.4 [11–19]	9.81	0.0074	−0.26	0.797
*t*_2_	16.6 ± 4.1 [8–26]	14.1 ± 3.2 [8–20]	13.8 ± 2.2 [11–17]	9.65	0.0080	0.24	0.816
*t*_3_	18.5 ± 4.3 [13–26]	14.3 ± 3.5 [8–20]	13.8 ± 2.2 [12–17]	5.05	0.080	0.41	0.691
*t*_4_	–	14.4 ± 2.2 [13–18]	12.0	2.42	0.120	2.45	n.a.
Disease duration/years							
*t*_0_	n.a.	9.4 ± 5.8 [1.9–32.4]	9.4 ± 4.2 [3.1–18.5]	0.15	0.699	−0.02	0.983
*t*_1_	n.a.	10.5 ± 5.8 [2.8–33.5]	10.4 ± 4.2 [4.0–19.4]	0.02	0.881	0.03	0.978
*t*_2_	n.a.	10.5 ± 7.3 [4.1–34.5]	11.7 ± 4.3 [5.7–19.6]	2.89	0.089	−0.54	0.593
*t*_3_	n.a.	11.6 ± 9.3 [5.1–35.4]	14.0 ± 4.4 [9.3–20.6]	0.50	0.480	−0.68	0.507
*t*_4_	n.a.	16.6 ± 13.5 [6.1–36.4]	11.9	0	1	0.70	n.a.
CERAD/total score							
*t*_0_	–	97.1 ± 5.2 [85.0–105.0]	95.2 ± 5.8 [86.0–106.0]	1.75	0.186	1.39	0.170
*t*_1_	–	98.4 ± 6.3 [81.0–108.0]	93.9 ± 6.7 [84.0–105.0]	0.54	0.464	2.71	0.0092
*t*_2_	–	97.8 ± 8.3 [77.0–105.0]	96.9 ± 6.0 [90.0–109.0]	1.18	0.278	0.29	0.774
*t*_3_	–	97.8 ± 8.0 [78.5–107.0]	91.5 ± 10.5 [75.5–102.0]	0.10	0.747	0.81	0.432
*t*_4_	–	102.5 ± 5.1 [95.0–106.0]	101.0	0.50	0.480	0.59	n.a.
PANDA/score							
*t*_0_	22.4 ± 5.9 [8–29]	21.9 ± 4.8 [15–30]	23.2 ± 4.9 [13–28]	5.52	0.019	−0.79	0.438
*t*_1_	22.1 ± 5.9 [9–29]	22.3 ± 5.8 [12–30]	24.0 ± 4.7 [16–27]	0.48	0.488	−1.01	0.322
*t*_2_	24.1 ± 3.4 [19–30]	20.8 ± 7.5 [10–30]	22.2 ± 7.6 [12–29]	0.06	0.800	−0.32	0.759
*t*_3_	20.1 ± 6.9 [10–29]	23.0 ± 3.3 [19–29]	19.0 ± 8.5 [11–28]	2.97	0.085	0.79	0.506
*t*_4_	–	25.7 ± 4.9 [20–29]	26.0	n.a.	n.a.	−0.12	n.a.
MMSE/score							
*t*_0_	29.3 ± 1.0 [26–30]	29.1 ± 1.1 [27–30]	28.6 ± 1.2 [26–30]	9.14	0.010	1.60	0.115
*t*_1_	29.3 ± 0.9 [26–30]	29.0 ± 1.1 [25–30]	28.7 ± 1.3 [25–30]	6.87	0.032	0.86	0.396
*t*_2_	29.3 ± 0.9 [26–30]	28.9 ± 1.3 [26–30]	29.4 ± 0.9 [28–30]	4.00	0.135	−1.29	0.209
*t*_3_	29.9 ± 0.3 [29–30]	28.6 ± 2.1 [26–30]	29.4 ± 0.9 [28–30]	3.90	0.142	−0.75	0.468
*t*_4_	–	29.0 ± 1.4 [27–30]	–	n.a.	n.a.	n.a.	n.a.
Hoehn&Yahr/Stage							
*t*_0_	n.a.	2.2 ± 0.6 [1.0–4.0]	2.2 ± 0.7 [1.0–4.0]	0.12	0.734	0.28	0.784
*t*_1_	n.a.	2.3 ± 0.7 [1.0–4.0]	2.2 ± 0.7 [1.0–4.0]	1.36	0.244	0.63	0.530
*t*_2_	n.a.	2.1 ± 0.7 [1.0–3.0]	2.5 ± 0.9 [1.0–4.0]	0.39	0.531	−1.30	0.210
*t*_3_	n.a.	2.3 ± 0.7 [1.0–3.0]	2.8 ± 0.7 [2.0–4.0]	0.13	0.720	−1.37	0.191
*t*_4_	n.a.	2.2 ± 0.8 [1.0–3.0]	1.0	1.50	0.221	3.21	n.a.
UPDRS III/score							
*t*_0_	n.a.	17.2 ± 8.7 [5–45]	17.8 ± 6.3 [6–30]	0.31	0.578	−0.29	0.770
*t*_1_	n.a.	18.8 ± 8.5 [3–41]	21.5 ± 10.4 [4–51]	0.62	0.431	−1.11	0.274
*t*_2_	n.a.	19.9 ± 8.2 [8–36]	21.1 ± 10.9 [3–34]	0.20	0.654	−0.32	0.756
*t*_3_	n.a.	21.6 ± 6.9 [13–38]	26.1 ± 8.3 [10–36]	0.05	0.828	−1.24	0.237
*t*_4_	n.a.	21.8 ± 12.2 [7–35]	7	1.41	0.235	2.70	n.a.

*Note: t*_0_: baseline, *t*_*n*_ *n*th‐year follow‐up. Values are given as mean ± *SD* [min–max]. *χ* ^2^ with corresponding *p*‐values refer to Kruskal–Wallis analysis of variances (ANOVA) across cognitively unimpaired PD patients that maintain their cognitive status throughout the study (nPD‐N), cognitively unimpaired PD patient that converted to either MCI or dementia on average after 1.4 years (cPD‐CI), and controls for each time point. Two right most columns refer to differences between PD patients (overall cohort) and controls are computed from unpaired *t* tests for continuous variables and ^+^Fisher's exact test for categorical variables for each time point.

**Table 5:** Volumetric results from atlas‐based volumetry (ABV) at study entry between “converters” and “nonconverters”

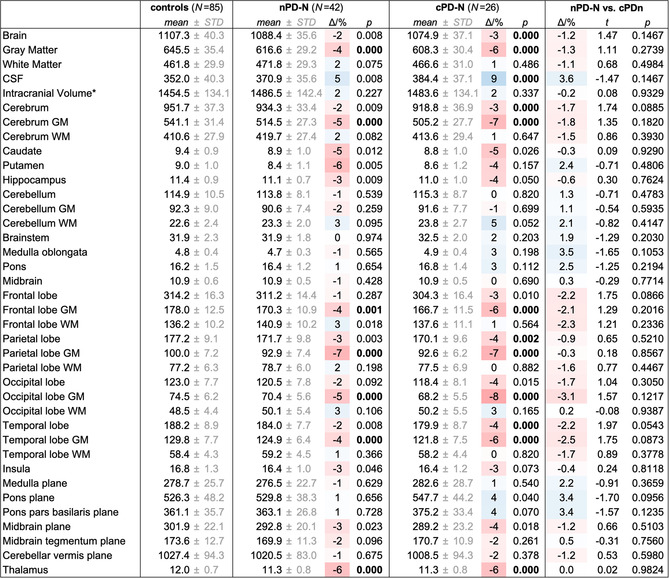

*Note*: Values are shown as volumes/cm^3^ and area/mm^2^ for planes. All values (*with the exception of the intracranial volume) are normalized to the study‐mean intracranial volume (1,459.3cm^3^) and adjusted to both the mean age (66.7 years) and mean years of education (14.3 years) of the whole study population at study entry. The given *p*‐values resulted from pair‐wise group comparisons between groups, that is, cognitively normal PD patients that maintain cognitive status throughout the study (“nonconverter,” nPD‐N), cognitively normal PD that converted (cPD‐N) to either MCI or dementia after 1.4 years on average, and healthy controls. Bold *p*‐values indicated statistical significance after Bonferroni correction for multiple testing. Deviation of means between groups are given as Δ/% = (*V*/*V*_controls_ − 1) × 100% and overlaid on a three‐color scale from shades of red (volume loss) over white (no change) to shades of blue (e vacuo volume gain). CSF, cerebrospinal fluid; GM, gray matter; WM, white matter.

#### 3.3.2 Results of cortical thickness measurements

**Figure 4:**
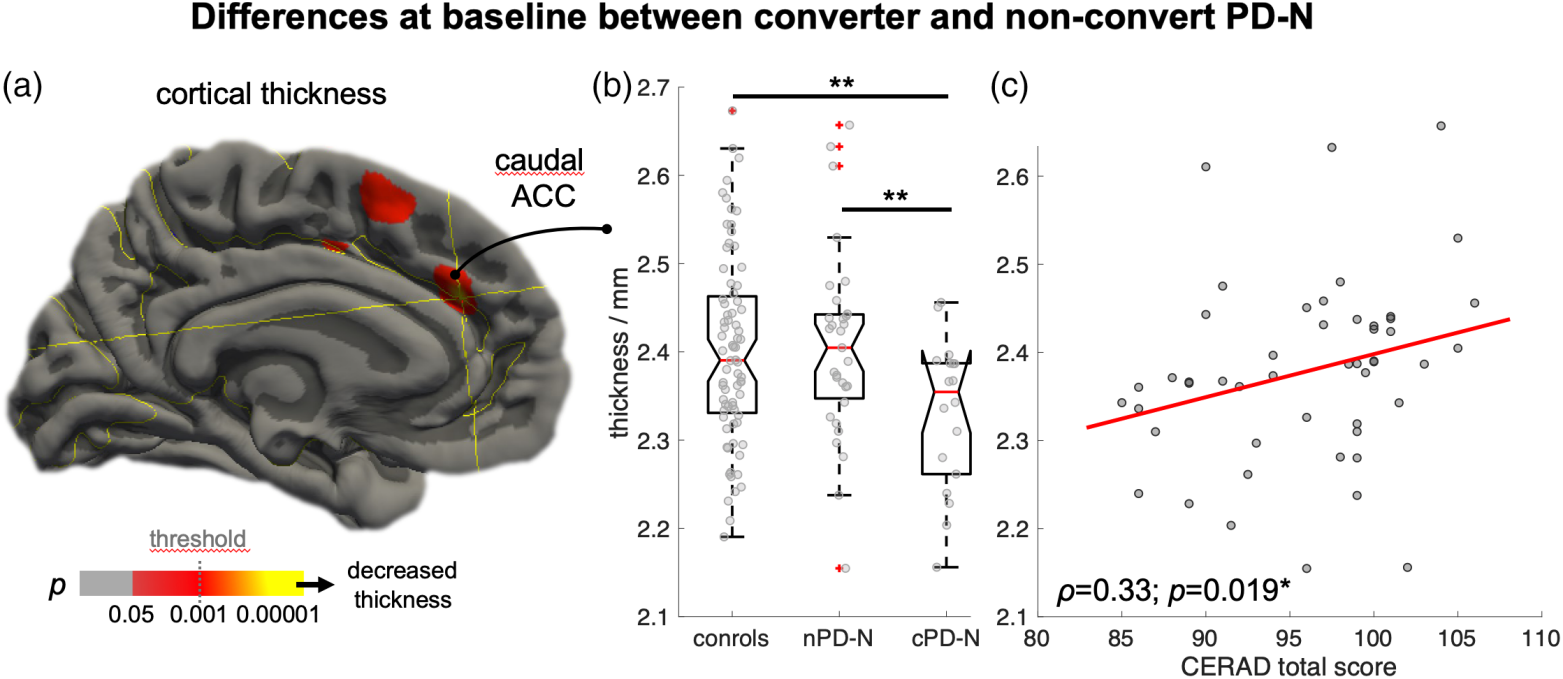
Differences in PD converters at study entry. (a) Vertex‐wise analysis showing the difference in cognitively unimpaired PD (PD‐N) at study entry between those PD‐N individuals who maintained “stable” cognition (nPD‐N, *n* = 31) and individuals prior 1.4 years on average before “conversion” (cPD‐N, *n* = 18) to either MCI or dementia. Cortical thinning in cPD‐N compared to nPD‐N is shown in hot colors (*p* < .001). (b) Region‐of‐interest‐based comparison between averaged cortical thickness values for both hemispheres revealed significant thinning in the caudal anterior cingulate cortex (ACC) in cPD‐N individuals as compared to both nPD‐N (*p* = .009) and controls (*p* < .005). (c) Caudal ACC thickness in PD‐N individuals at study entry was significantly correlated with the CERAD total score (*p* = .019)

Explorative vertex‐wise analysis suggested cortical thinning mainly in the anterior cingulate cortex (ACC) in cPD‐N patients compared to controls (*p* < .001, uncorrected) at baseline (Figure 4a). Region‐of‐interest‐based comparison of cortical thickness data confirmed significant cortical thinning in the caudal ACC as indicated by Kruskal–Wallis ANOVA for nPD‐N, cPD‐N, and controls (*χ* ^2^ = 6.22, *p* = .045). The significant effect across groups resulted from a significant thinning in cPD‐N as compared to both nPD‐N (−3% thickness reduction, post hoc *t* = −2.72, *p* = .0095) and controls (−3%, post hoc *t* = −3.04, *p* = .0048) but not from differences between nPD‐N and controls (post hoc *t* = 0.18, *p* = .857), as shown in Figure 4b. The caudal ACC thickness measures for PD‐N (values pooled from nPD‐N and cPD‐N) were significantly correlated with the CERAD total score (*r* = .33, *p* = .019) as shown in Figure 4c. Region‐of‐interest analysis of all other regions revealed no significant effects between nPD‐N and cPD‐N. Overall, the difference between nPD‐N and cPD‐N resulted exclusively from differences in ACC thickness at study entry but neither from volumetric or thickness changes in other brain regions nor from different atrophy progression rates throughout the observational period.

## 4 DISCUSSION

Using high‐resolution MRI data from the LANDSCAPE study cohort collected over a four‐year observational period to study PD‐associated atrophy progression, we demonstrated a longitudinal pattern of brain atrophy and cortical thinning in PD. The patients with a disease duration of about 9 years on average at study entry could be considered as “advanced” PD patients given that about 50% of all PD patients develop dementia within 10 years of diagnosis (Anang et al., 2014). Volumetric alterations were already present at study entry in both cognitively normal PD and in cognitively impaired PD individuals. Longitudinal analysis of the 597 data sets from fully neuropsychologically characterized individuals with PD and healthy age‐matched controls suggested that atrophy progression in advanced PD is similar as in the “normal” aging brain. These results were obtained from a LME model that also incorporates the nonlinear effect of “normal” brain aging which is characterized by a small rate of atrophy change under 60 years but a gradually accelerated pattern of “normal” brain atrophy progression over 60 years of age—a finding that is fully consistent with a meta study of volumetric MRI data from studies in healthy elderlies (Hedman et al., 2012).

We demonstrated almost the same atrophy progression rate in our PD cohort regardless of the cognitive status which is apparently at odds with previous longitudinal studies in PD that reported accelerated atrophy progression in PD (Mak et al., 2015; Yau et al., 2018)—the main difference is, however, that these studies enrolled newly diagnosed PD or de‐novo PD patients. Our data were generated within the LANDSCAPE study aiming at identifying factors which contribute to both the evolution and progression of cognitive impairment in PD (Balzer‐Geldsetzer et al., 2011) so that patients with and without dementia were eligible for enrolment which resulted in a cohort of advanced patients. Whereas the rates of volume loss and regional cortical thickness change over time were almost identical among patient groups relative to controls, there was moderately more accelerated thinning of the overall cortical thickness in PD‐N relative to PD‐CI patients and healthy controls. This overall accelerated cortical thinning in PD‐N patients which were “earlier” in the disease course (as supported by the lower disease duration) suggests the straightforward conclusion that brain atrophy beyond that of healthy aging occurs early in the course of PD while it does not differ from controls even when the cognitive functions decrease. In accordance with these findings, cross‐sectional analysis at study entry demonstrated a widely distributed pattern of damage (atrophy) involving subcortical areas including the lower brainstem and striatum but also prefrontal, temporal, parietal and occipital lobe, as previously described in other studies (Zarei et al., 2013). The observed atrophy distribution in PD is consistent with staging (Braak et al., 2003; Braak & Del Tredici, 2009): the disease progression up to this point (of enrolment) has led to a considerable damage to the brain caused by the underlying pathological process that was accordingly demonstrated in the subgroup of cognitively normal PD patients. It appears that a barely detectable threshold of “decompensation” for cognitive performance has been crossed soon after the neuropsychologically overt phase of MCI (Stern, 2012). Gradual worsening of cognitive performance is best explained by the damage to specifically eloquent brain areas rather than the rate of atrophy progression—a statement which is consistent with the concept that pathology progresses for years before initial symptoms become clinically recognizable (Braak & Del Tredici, 2017). Accelerated atrophy progression early in the disease course leads to a considerable neuronal damage of cognition‐related brain areas with early involvement of memory‐related areas including temporal lobe and hippocampal regions. By the time that a patient begins to experience the initial cognitive problems, the condition has been already well established in the brain (Braak, Rüb, Jansen Steur, Del Tredici, & de Vos, 2005). Up to some point, redundant capacities are almost exhausted (Stern, 2006) such that a gradual but normal atrophy progression rate in the predamaged brains leads to MCI (Kalbe et al., 2016).

To what extent this threshold is dependent on the regional pattern of atrophy distribution has been addressed by the comparison between cognitively normal PD “converters” and “nonconverters.” The results suggested that cognitive decline within the observational period, that is, a “cognitive performance” conversion from PD‐N to either MCI or PDD after about 1.4 years on average, might be predicted by regional cortical thinning in the caudal portion of the anterior cingulate cortex (ACC). Thinning in the ACC in the cognitively normal status of PD possibly predicts MCI in the nearby future whereas the atrophy progression rate (which was similar among groups) appears to have no impact on predicting MCI. The ACC which is a core hub of the limbic system, presented some degree of predictability for upcoming cognitive decline affecting executive, attentional, visuospatial, and memory domains, a result that was strengthened by the correlation with cognitive performance. The burden and distribution of the lesion pattern is best explained by staging (Braak et al., 2003; Braak & Del Tredici, 2009), according to which the involvement of the ACC is assigned to neuropathological disease stage four while the pathological process enters the neocortices in stage five. The present study sheds light on the yet unmet urgent need to identify imaging biomarkers aiming at identifying patients at risk for developing cognitive deficits in PD (Delgado‐Alvarado, Gago, Navalpotro‐Gomez, Jiménez‐Urbieta, & Rodriguez‐Oroz, 2016): thinning in the ACC in a “cognitively normal” PD individual appears to be a promising candidate for providing reliable prognostic information. Identifying those patients at risk to convert to the status of MCI is particularly important for stratifying PD patients for future clinical studies (Lanskey et al., 2018).

With respect to methodological considerations on 3D MRI data processing and longitudinal data analysis in the current study, absolute volumetric assessment like ABV, a fully automated and highly reproducible approach, may be promising to accurately capture PD‐related anatomical alterations (Huppertz et al., 2016). In a different MRI parameter approach, cortical thickness measurements emerged as a valuable measure of brain morphometrics in the cortex that are sensitive to age‐ and disease‐associated gray matter alterations (Fortin et al., 2018); Yau et al., 2018). Here, we used LME models as a more powerful and versatile framework for the longitudinal analysis of volumetric and cortical thickness data (Fitzmaurice & Ravichandran, 2008). The LME models are preferential to properly handle unbalanced longitudinal data in the elderly PD patients, that is, a different number of measurements for each individual due to drop outs and time variability around the scheduled date for follow‐up assessments and allow also to include data sets with a single measurement in order to add value to inter‐subject variation (Fitzmaurice, Laird, & Ware, 2011). Piecewise modeling of age‐dependence on both regional brain volumes and cortical thickness can be accomplished using LME and is required since a steady and gradual volume loss and cortical thinning has been consistently demonstrated for the aging brain in healthy elderly over 60 years (Hedman et al., 2012).

The current study is not without limitations. The molecular mechanisms leading to morphometric brain alterations in neurodegeneration are not fully understood, nevertheless, volumetric and thickness measures as obtained from high‐resolution MRI have been shown to reflect changes in density and most likely are the result of tissue loss due to the neurodegenerative process in PD (la Fougère et al., 2011). At present there are no techniques available that allow a quantitative measure of both the load and the precise location of misfolded α‐synuclein in the brain. We can only capture the probable consequence of the process, that is, volume loss and cortical thinning, but not the possibly complex relation between misfolded α‐synuclein accumulation and these changes. One major limitation of our study is that the neuropsychological classification of PD‐N and PD‐MCI was not based on the recently proposed MDS criteria (Litvan et al., 2012) as these diagnostic criteria for PD‐MCI were not available at study setup (Balzer‐Geldsetzer et al., 2011). Due to the fact that the diagnosis of PD‐MCI in our study only requested one cognitive test to be below the cut‐off for cognitive impairment, while the MDS criteria request at least two cognitive tests to be impaired, the number of PD patients with CI might be overestimated in our study. However, with the more conservative classification (two tests below cut‐off) and thus possibly less “false positive” patients with MCI or, in other words, a more homogeneous group with clear CI, it could be expected that the results presented here regarding brain atrophy would even come out in a more pronounced way. This notion will have to be subject of further research. Although our sample from the LANDSCAPE is large, it may not be an epidemiologically representative cohort due to a research‐oriented selection bias, and the MRI study is limited by the attrition rate with a drop‐out of follow‐up investigations due to increasing physical and mental disability (details in Figure S1). The sample of individuals with full‐blown dementia is relatively small as compared to the MCI cohort and did not allow to carry out a robust separate longitudinal investigation. Given the well‐known variability of the MCI status, the pooling of PD‐MCI and PDD is a limitation of the study. Nevertheless, the present study does not intend to disentangle PD‐MCI and PDD from an imaging point of view. Finally, the study is not autopsy‐controlled and a possible coincidental occurrence of Alzheimer's disease in advanced PD cases with severe cognitive impairment may be missed (Berg et al., 2014; McMillan & Wolk, 2016).

This study is, to our best knowledge, the largest yet to longitudinally analyze MRI scans in neuropsychologically well‐characterized “advanced” PD patients. Whether cognitive deficits become overt may possibly depend on ACC involvement as an integrative structure. Finally, the longitudinal in vivo analysis of atrophy patterns in advanced PD has helped to shift towards greater awareness of an almost “normal” cortical atrophy progression rate indicating that severe neuronal damage most likely occurs early in course of PD. This study conclusion emphasizes the urgent need for potential causal therapeutic concepts as early as possible (Dehay et al., [Link]) before the natural brain compensation capacities may be exhausted by the underlying PD‐associated pathological process.

## Supporting information


**Figure S1** Flow chart.
**Table S1:** Basic site‐specific demographic features and MRI scanning protocols.

## Data Availability

The data that support the findings of this study are available from the corresponding author upon reasonable request.
