# Supplementary material for: Longitudinal brain atrophy distribution in advanced Parkinson's disease: What makes the difference in “cognitive status” converters?
Source: Hum Brain Mapp. 2019 Dec 2;41(6):1416–34. doi: 10.1002/hbm.24884 (PMC7267933; doi:10.1002/hbm.24884)
Supplement: Supplementary file 1 — Figure S1 Flow chart. Table S1: Basic site‐specific demographic features and MRI scanning protocols. [file HBM-41-1416-s001.docx]

**Supporting Information**

**Longitudinal brain atrophy distribution in advanced Parkinson’s disease:**

**What makes the difference in ‘cognitive status’ converters?**

Martin Gorges^1^, Martin S. Kunz^1^, Hans-Peter Müller^1^, Inga Liepelt-Scarfone^2^, Alexander Storch^3,4,5^, Richard Dodel^6,7^, Rüdiger Hilker-Roggendorf^8^, LANDSCAPE Consortium, Daniela Berg^2,9^, Elke Kalbe^10^, Heiko Braak^1^, Kelly Del Tredici^1^, Simon Baudrexel^11^, Hans-Jürgen Huppertz^12^, Jan Kassubek^1^

^1^Department of Neurology, University of Ulm, Ulm, Germany

^2^German Center of Neurodegenerative Diseases and Hertie Institute for Clinical Brain Research, Tübingen, Germany

^3^Department of Neurology, University of Rostock, Rostock, Germany.

^4^Division of Neurodegenerative Diseases, Department of Neurology, Technische Universität Dresden, Dresden, Germany

^5^German Centre for Neurodegenerative Diseases (DZNE) Rostock/Greifswald, 18147 Rostock, Germany

^6^Department of Neurology, Philipps University Marburg, Marburg, Germany

^7^ Department of Neuro-Geriatrics, University Clinic, Essen, Germany

^8^Klinik für Neurologie und Klinische Neurophysiologie, Klinikum Vest, Knappschaftskrankenhaus Recklinghausen, Recklinghausen, Germany

^9^Department of Neurology, Christian Albrecht University, Kiel, Germany

^10^Medical Psychology | Neuropsychology and Gender Studies, Center for Neuropsychological Diagnostics and Intervention (CeNDI), University Hospital Cologne, Cologne, Germany

^11^Department of Neurology, J.W. Goethe University, Frankfurt/Main, Germany

^12^Swiss Epilepsy Centre, Klinik Lengg, Zürich, Switzerland

**METHODS**

**SI Figure 1: Flow chart.**


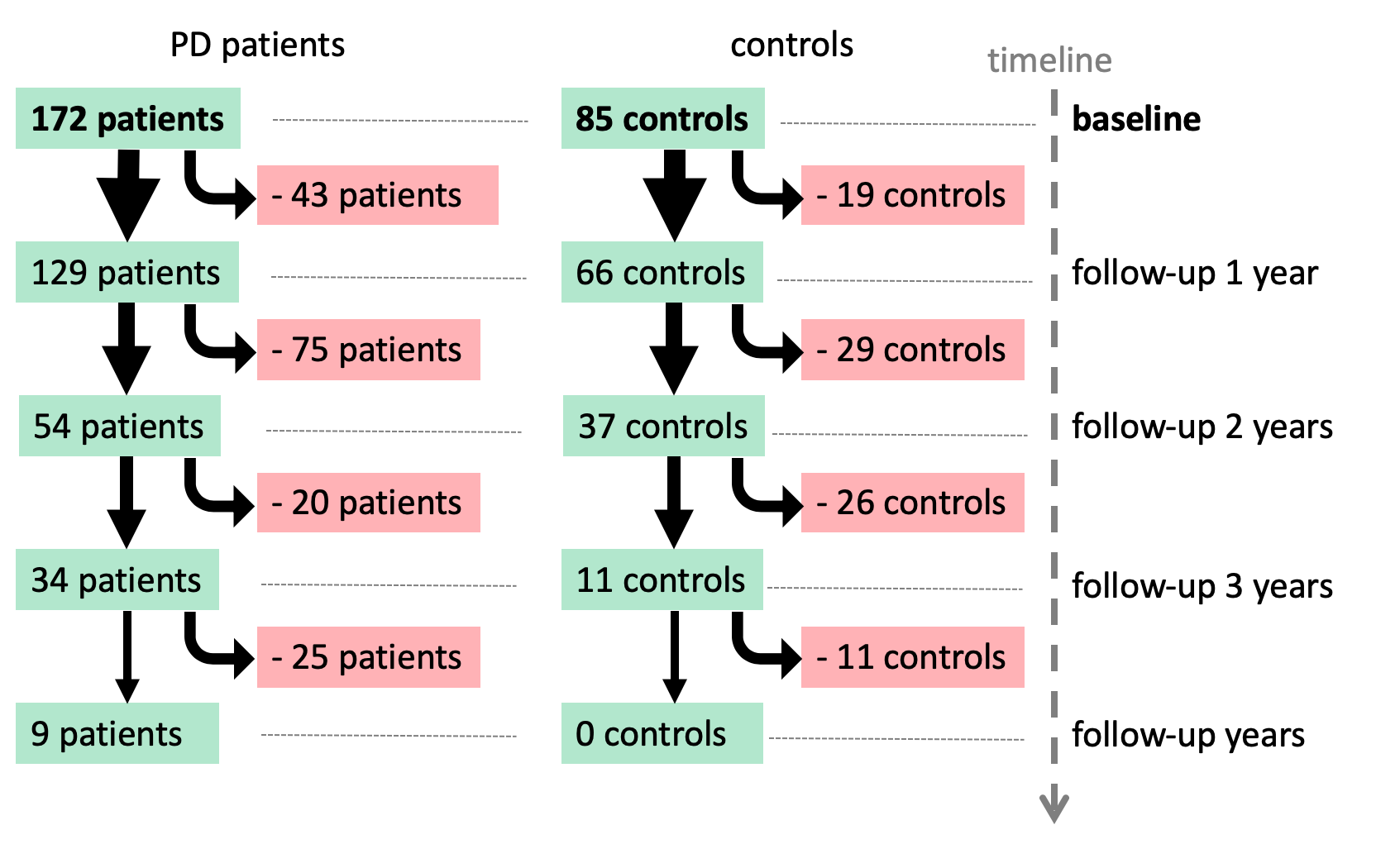


Flow chart indicating the number of drop outs of PD patients (left) and healthy controls (right) during the study. The attrition rate was mainly due to increasing disease burden. The resulting imbalanced number of individuals per timepoint required appropriate data modelling as performed with the linear mixed effect approach.

**SI Table 1: Basic site-specific demographic features and MRI scanning protocols.**

| **Centre** | **01** | **02** | **03** | **04** | **05a** | **05b** | **06** |
| --- | --- | --- | --- | --- | --- | --- | --- |
| PD data sets | 53 | 59 | 54 | 46 | 53 | 28 | 105 |
| HC data sets | 21 | 52 | 31 | 25 | 34 | 12 | 24 |
| Vendor | SIEMENS | SIEMENS | SIEMENS | SIEMENS | SIEMENS | SIEMENS | PHILIPS |
| Model | Verio | Trio | TrioTim | TrioTim | Allegra | Prisma | Achieva |
| Field/T | 3.0 | 3.0 | 3.0 | 3.0 | 3.0 | 3.0 | 3.0 |
| TR/ms | 2500 | 2500 | 2500 | 2500 | 2500 | 2500 | 7.3/6.9 |
| TE/ms | 4 | 4 | 4 | 4 | 4 | 2 | 3 |
| Voxel (x/y/z)/mm^3^ | 1.0/1.0/1.0 | 1.0/1.0/1.0 | 1.0/1.0/1.0 | 1.0/1.0/1.0 | 1.0/1.0/1.0 | 1.0/1.0/1.0 | 1.0/1.0/1.0 |

Parkinson’s disease (PD) patients and healthy controls from six German sites (Dresden, Frankfurt, Marburg, Tübingen, Ulm, and Kiel). The scanner for site Ulm ‘05’was replaced resulting in two different protocols. TR=time repetition, TE=time echo.
